# Condensation of Counterions Gives Rise to Contraction Transitions in a One-Dimensional Polyelectrolyte Gel

**DOI:** 10.3390/polym10040432

**Published:** 2018-04-13

**Authors:** Gerald S. Manning

**Affiliations:** Department of Chemistry and Chemical Biology, Rutgers University, 610 Taylor Road, Piscataway, NJ 08854-8087, USA; jerrymanning@rcn.com; Tel.: +1-617-332-7037

**Keywords:** polyelectrolyte gel, bead-spring chain, swelling, counterion condensation, elasticity

## Abstract

The equilibrium volume of a polyelectrolyte gel results from a balance between the tendency to swell caused by outbound polymer/counterion diffusion along with Coulomb interactions on the one hand; and, on the other, the elastic resilience of the cross-linked polymer network. Direct Coulomb forces contribute both to non-ideality of the equilibrated Donnan osmotic pressure, but also to stretching of the network. To isolate the effect of polyelectrolyte expansion, we have analyzed a “one-dimensional” version of a gel, a linear chain of charged beads connected by Hooke’s law springs. As in the range of weak Coulomb strengths previously studied, the springs are significantly stretched by the repulsive interactions among the beads even when the Coulomb strength is strong enough to cause condensation of counterions. There is a quasi-abrupt transition from a stretched state to a partially collapsed state in a transition range between weak and strong Coulomb strengths. Fluctuations between stretched and contracted conformations occur within the transition range. As the solvent quality decreases past the transition range, a progressive collapse can result if the condensed counterions strengthen the spring constant.

## 1. Introduction

The swelling and deswelling transitions of gels have long been the subject of wide-ranging experimental and theoretical research, motivated both by a large number of practical applications but also by the intrinsic interest in gels as systems subject to the fundamental physical laws of phase transitions [1,2,3,4,5,6,7,8,9]. Gels can swell in good solvents, but they also collapse progressively or discontinuously depending on their chemical composition and environmental factors.

A gel is a cross-linked polymer network. The elastic resilience of the network resists outward polymer diffusion, and the balanced structure at equilibrium has a volume less than the volume of its container, and hence a polymer density greater than in a solution of free chains uniformly filling the container. In the case of a macroscopic polyelectrolyte gel, the polymers are accompanied by their counterions to maintain electroneutrality inside the gel. However, in addition, the polymer chains are stretched by direct Coulomb charge repulsion, contributing toward the equilibrated volume. Chain-chain repulsion can also contribute [10], but the role in gels of loose clustering of polymers mediated by condensed univalent counterions has yet to be assessed [11,12,13,14,15]. Stretching of polyelectrolyte chains by Coulomb repulsions of cross-links appears in simulations as the dominant reason for the swelling of ionized nanogels beyond the radius of the volume attained when electrostatic forces are negligible [8]. Our current thinking is that direct Coulomb stretch, whether by interactions among the points of cross-linking or among the charged groups along the chains themselves, may be important even in macroscopic gels.

In this paper, we continue the study of an ionized bead-spring chain as a “one-dimensional gel” begun previously [16]. Nearest-neighbor beads are connected by identical springs obeying Hooke’s law. The unstressed springs have zero length; the unstressed chain would therefore be completely collapsed. However, each bead is electrostatically charged, and Coulomb repulsions among beads place stress on the springs, which stretch to some equilibrium length. We take the equilibrium spring length $b_{\mathrm{eq}}$ as a measure of swelling of the “gel”. In this model, “swelling” is due entirely to direct Coulomb repulsion as resisted by the elasticity of the springs.

Our previous paper on the bead-spring chain was restricted to weak Coulomb strengths. Specifically, the equilibrium spring lengths, or, equivalently, the equilibrium charge spacing on the chain, was always greater than the Bjerrum length (defined below). In this range, there is no counterion condensation, and Debye-Hückel electrostatics provides an accurate account of the bead-bead repulsions [17,18]. The primary results there for present purposes were quantitative verifications of the expectations, first, that the equilibrium gel should indeed be swollen, with $b_{\mathrm{eq}}>0$; second, that the degree of swelling should be less for stiffer springs; and, third, that the degree of swelling should increase with Coulomb strength, that is, $b_{\mathrm{eq}}$ should become greater for lower temperatures and/or lower dielectric constants (poorer solvent quality). At the cost of additional modeling, we also found that the radius of gyration, another measure of swelling, increases as the solvent becomes poorer. The observed behavior in poorer solvents is expected with Debye-Hückel interactions, which do not draw counterions in toward the chain very strongly, and therefore only partially counteract the increased bead-bead repulsions in conditions of increased Coulomb strength. With Debye-Hückel interactions, the gel is always swollen, and there is never a collapse.

In this paper, we consider the range of strong Coulomb interactions where the equilibrium spring length is smaller than the Bjerrum length, and counterions condense on the chain. We will see that the weak and strong Coulomb regimes overlap, and interesting behavior in the overlap region is observed. We will then introduce additional assumptions, qualitatively similar to ion-pairing, that allow the bead-spring assembly to contract toward small values of the spring length.

## 2. Definitions and Free Energy for Strong Coulomb Interactions

We consider a line of identical point charges (beads). The charges are each equal to *e* (polycation) or to −e (polyanion), where *e* is the positive unit charge. The bead spacing is uniformly equal to *b* (unsigned bare charge density e/b). Consecutive beads are connected by identical Hooke’s law springs with spring constant *a*. This “one-dimensional gel” is then a line of identical charge sites with nearest neighbor sites attached with springs, all instantaneously of the same but variable length.

We take the unstressed lengths of all springs as zero. In other words, we assume that the gel would be completely collapsed in the absence of charges on the beads. However, because the springs are stretched by the mutual repulsions of the charged beads, their common equilibrium length, which we call $b_{\mathrm{eq}}$, will not be zero. The equilibrium spring length is a measure of the extent of gel swelling. The value of $b_{\mathrm{eq}}$ will depend on both mechanical and electrostatic characteristics, such as the spring constant *a* and the dielectric constant of the solvent. For example, we expect larger values of *a* to produce smaller values of $b_{\mathrm{eq}}$, since stiffer springs should be stretched less by given electrostatic repulsions.

The beads on the chain interact electrostatically with one another. The bare free energy of interaction between a pair of beads separated by distance nb along the chain is given by $k_{B}Tl_{B}/nb$, where $k_{B}$ is Boltzmann’s constant, *T* is Kelvin temperature, and $l_{B}$ is the Bjerrum length of the solvent. To give Coulomb’s law for the bare bead-bead interaction in the solvent, the Bjerrum length is defined by $l_{B}=e^{2}/4\pi \epsilon_{0}\epsilon k_{B}T$, where $\epsilon_{0}$ is the vacuum permittivity, and ϵ is the temperature-dependent dielectric constant of the pure solvent.

As in the previous paper [16], the Bjerrum length plays a large role here. It can be characterized as the distance at which two isolated unit charges have Coulomb interaction free energy equal to $k_{B}T$. The interaction is weaker than $k_{B}T$ for greater separations, and stronger than $k_{B}T$ if the charges are closer. This characterization follows by setting the bead-bead separation nb in the previous paragraph equal to $k_{B}T$. The Bjerrum length can be said therefore to measure the Coulomb strength of the solvent, in that the Coulomb interaction of charges in a solvent of greater Bjerrum length extends to longer separation distances before being overcome by thermal disorder. Greater Bjerrum lengths at a given temperature are achieved by solvents of lower dielectric constants, so a large Bjerrum length is associated with a poor solvent for ions.

The previous paper was restricted to relatively low bare charge densities; the equilibrium charge spacings $b_{\mathrm{eq}}$ had values greater than $zl_{B}$, where *z* is the unsigned valence of the counterions. In this range, counterion condensation does not occur, and Debye-Hückel screened Coulomb potentials are an accurate description of the electrostatic interactions among beads [17,18]. This paper focuses on high charge densities, where $b_{\mathrm{eq}}<zl_{B}$, and counterion condensation maintains the net charge density as one net charge in length $zl_{B}$. In a simple picture, the condensed counterions are mobile along the chain, and the electrostatic component of the free energy includes not only the reduction of net charge, but also the translational entropy of the condensed counterions, and the determination of the local volume containing the condensed counterions [19]. The expression for the overall free energy $g_{\mathrm{cc}}$ of the bead-spring assembly per bead in units of $k_{B}T$ is then given by,

(1) $$g_{\mathrm{cc}}=\frac{1}{2}ab^{2}-\frac{1}{z}\left(2-\frac{b}{zl_{B}}\right)\ln\left(1-e^{-\kappa b}\right)-\frac{1}{z}+\frac{b}{z^{2}l_{B}}$$

The first term is the spring energy, where the units of the reduced spring constant *a* are inverse square length, and the last three terms together give the electrostatic contribution (which is nonlinear in counterion condensation theory [19]). We have taken the chain as consisting of *N* beads connected by N−1 springs with N>>1, so that N−1≈N, and the length of the chain $Nb>>\kappa^{-1}$. In other words, we take the chain as sufficiently long to justify neglect of end effects. The inverse screening length κ is defined by $\kappa^{2}=4\pi l_{B}\sum_{i}c_{i}z_{i}^{2}$, $c_{i}$ representing the bulk concentration (number of ions per m${}^{3}$) of small ions of species *i*, and $z_{i}$ their unsigned valence. Finally, the subscript on $g_{\mathrm{cc}}$ for “counterion condensation” distinguishes it from $g_{\mathrm{dh}}$, the corresponding Debye-Hückel free energy of the previous paper for low charge densities and no counterion condensation. We will make use of $g_{\mathrm{dh}}$ at a subsequent point of this paper.

The equilibrium spring length $b_{\mathrm{eq}}$ is found as the root of the condition $\partial g_{\mathrm{cc}}/\partial b=0$ for fixed values of the other parameters $\left\{z,a,l_{B},\kappa \right\}$ of $g_{\mathrm{cc}}$. The spring length then emerges as a function of these other quantities. In this paper, as in the previous one, we focus on the dependence of $b_{\mathrm{eq}}$ on spring constant *a* and Bjerrum length (Coulomb strength) $l_{B}$, the latter as a measure of solvent quality. We recognize also that at fixed ionic strength, κ varies with $l_{B}$.

## 3. The Equilibrium Spring Length is Shorter for Stiffer Springs

The free energy $g_{\mathrm{cc}}$ contains two positive terms, the mechanical energy stored in the stretched springs, and the electrostatic free energy stored when the neutral polymer chain is charged. However, the dependencies on charge spacing *b* are not mutually reinforcing. When the springs are stretched, the mechanical spring energy increases, but the electrostatic free energy decreases (because the separations of charged beads increase). The equilibrium condition is $\partial g_{\mathrm{cc}}/\partial b=0$, and then the equilibrium value of the stretched spring $b_{\mathrm{eq}}$ is the root of this equation. In the previously treated case of weak Coulomb interactions [16], an increased spring constant resulted, as expected, in a decreased ability of the bead-bead charge repulsions to stretch the spring. In the case at hand, the condensed counterions act to screen the bead charges even more effectively than the relatively weak Debye-Hückel diffuse ion atmospheres, so we again expect stiffer springs to be stretched less by charge-charge repulsion.

**Figure 1:**
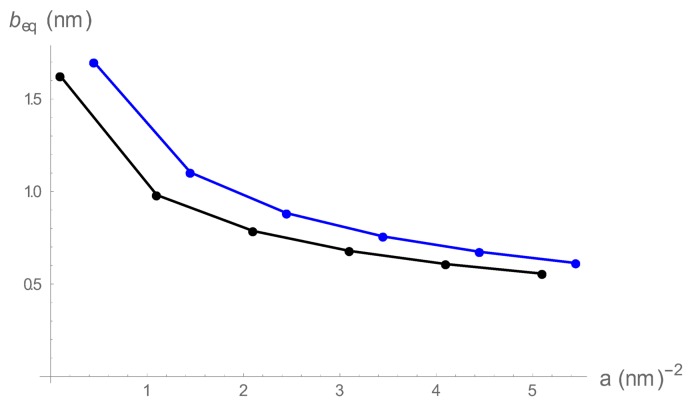
The equilibrium bead spacing as a function of spring constant in the counterion condensation range. The Bjerrum length is fixed at 1.7 nm, characteristic of methanol at room temperature. The 1:1 salt concentrations are 0.01 M (black), and 0.001 M (blue).

We give a numerical example in Figure 1, where the Bjerrum length is fixed at the value for methanol, $l_{B}=1.7$ nm at room temperature, at two different 1:1 salt concentrations, 0.01 and 0.001 M. We are in the condensed counterion region throughout the range shown, with $b_{\mathrm{eq}}$ always less than $l_{B}$ (equal to $l_{B}$ for the left-most point). The expectation is realized; the equilibrium springs become shorter as they become more stiff. Additionally, as expected, the “ swelling” is greater at lower salt concentration (where Coulomb repulsion among beads is stronger).

## 4. Contraction Fluctuations in a Transition Region

There is a transition region between weak and strong Coulomb interactions. In this region relatively stretched conformations with low charge density and no counterions condensed on the chain coexist with relatively contracted states of relatively high bare charge density but with condensed counterions. To investigate this transition region, we need the free energy $g_{\mathrm{dh}}$ with Debye-Hückel electrostatics [16] as well as Equation (1) for $g_{\mathrm{cc}}$,

(2) $$g_{\mathrm{dh}}=\frac{1}{2}ab^{2}-\frac{l_{B}}{b}\ln\left(1-e^{-\kappa b}\right)$$

The equation is valid only in the region of low charge density $b>l_{B}$ where there are no condensed counterions. Its implicit inclusion of the counterion valence *z* only as affecting the Debye screening constant κ can be distinguished from the explicit appearance of *z* in Equation (1), which handles the counterion condensation range of high charge density $b<l_{B}$.

**Figure 2:**
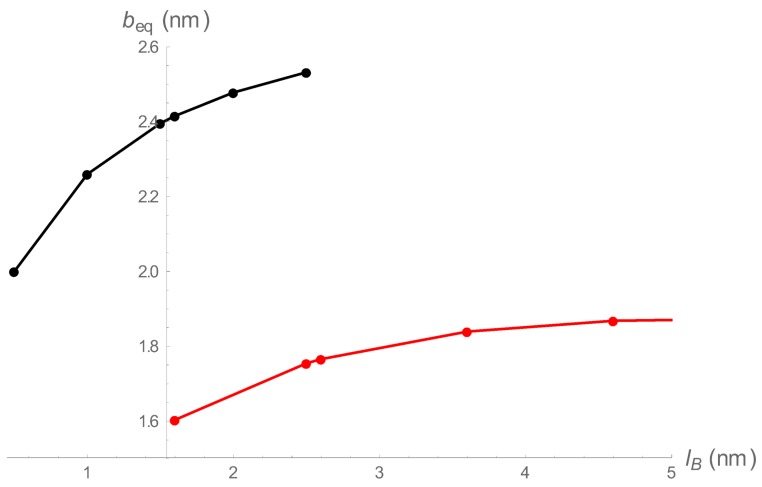
Equilibrium charge spacings as functions of Bjerrum length for low charge densities (black) and high charge densities (red), both at spring constant 0.1 (nm)${}^{-2}$ and 1:1 salt 0.01 M.

Figure 2 is a representative plot of $b_{\mathrm{eq}}$ as a function of Bjerrum length $l_{B}$ for both regions. The black points are roots of $\partial g_{\mathrm{dh}}/\partial b=0$, and the red points are roots of $\partial g_{\mathrm{cc}}/\partial b=0$. All points of the black curve satisfy $b_{\mathrm{eq}}>l_{B}$, and all points on the red curve satisfy $b_{\mathrm{eq}}<l_{B}$. For the conditions of Figure 2, there is an overlap range 1.60 nm $<l_{B}<$ 2.53 nm. For a given Bjerrum length in this range, there are two equilibrium spring lengths, a relatively stretched one (black) with no condensed counterions, and a relatively contracted one (red) with condensed counterions.

**Figure 3:**
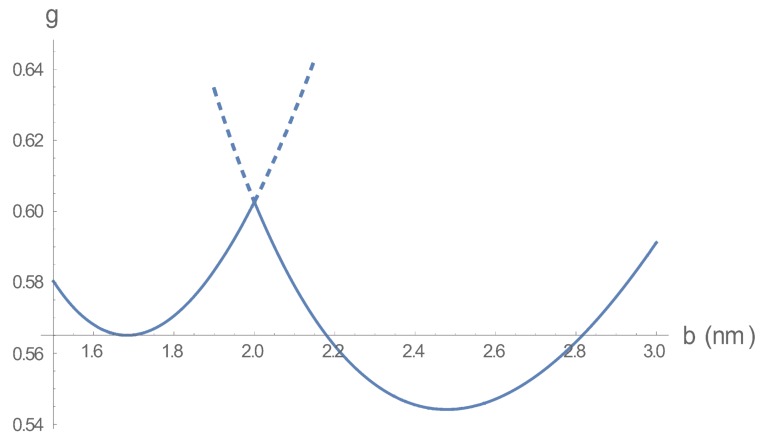
The free energy (per bead, in units of $k_{B}$*T*) as a function of bead spacing for a Bjerrum length *l*${}_{B}$ = 2.0 nm lying within the transition range in Figure 2; the spring constant and salt concentration are the same as in Figure 2. *g* = $g_{\mathrm{cc}}$(*b*) for *b* < 2.0 nm, g = $g_{\mathrm{dh}}$(*b*) for *b* > 2.0 nm. The dashed continuations are forbidden, so there is a maximum at *l*${}_{B}$ = 2.0 nm

To clarify this situation, we have constructed Figure 3 for the free energy as a function of bead spacing *b* for a value 2.0 nm of the Bjerrum length within the transition region. The two minima are located at the two $b_{\mathrm{eq}}$ values in Figure 2 at $l_{B}=2.0$ nm. The free energy profile follows the solid curve, since the dashed portions are forbidden; there, the values of *b* are less than 2.0 nm for $g_{\mathrm{dh}}$, and greater than 2.0 nm for $g_{\mathrm{cc}}$. The springs fluctuate in length between the two minima, the longer spacing representing a bead-spring assembly of relatively low charge density devoid of condensed counterions; the shorter one, a chain of relatively high bare charge density but with counterions condensed on it. The picture of the fluctuation could be that when counterions approach the chain, there is a stabilizing contraction followed by an expansion that releases the counterions.

**Figure 4:**
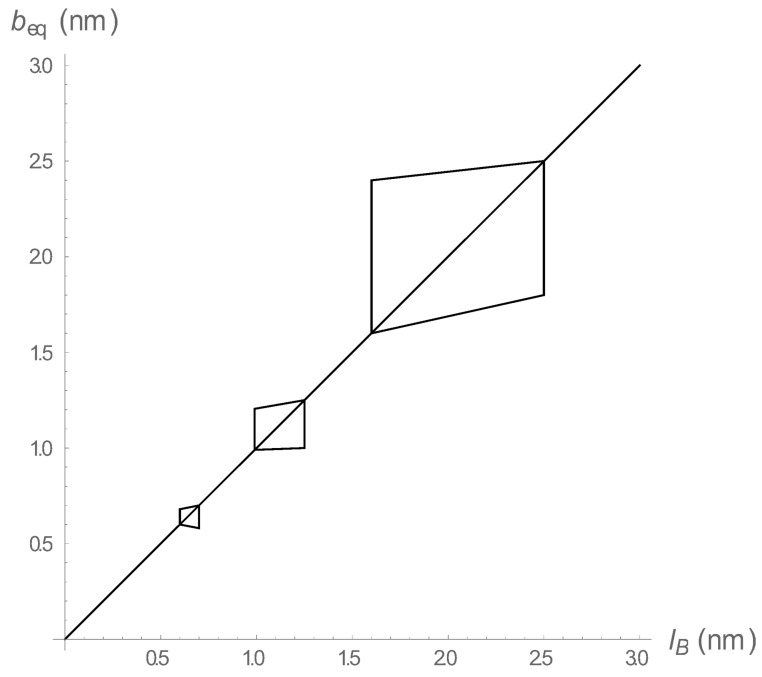
The fluctuation regime near the counterion condensation threshold. The three regions shown are, from left to right, for spring constants *a* = 5.0, 1.0, 0.1, all in (nm)${}^{-2}$. See text for further explanation.

Figure 2 and Figure 3 pertain to a fixed spring constant a=0.1 (nm)${}^{-2}$. In Figure 4 we show the shape of the transition regions for three distinct values of *a*. The fluctuations are greater for the weaker spring constant. If the spring constant is varied continuously, the transition regions continuously merge to form a funnel-shaped region centered on the line $b_{\mathrm{eq}}=l_{B}$, and tapering toward a point at the origin for very strong springs. Above and to the left of the funnel are equilibrium states of low-charge density stretched springs with no condensed counterions. Below and to the right are equilibrium states of contracted springs with condensed counterions present to ameliorate the high bare charge density.

## 5. Toward Collapse for High Coulomb Strength

**Figure 5:**
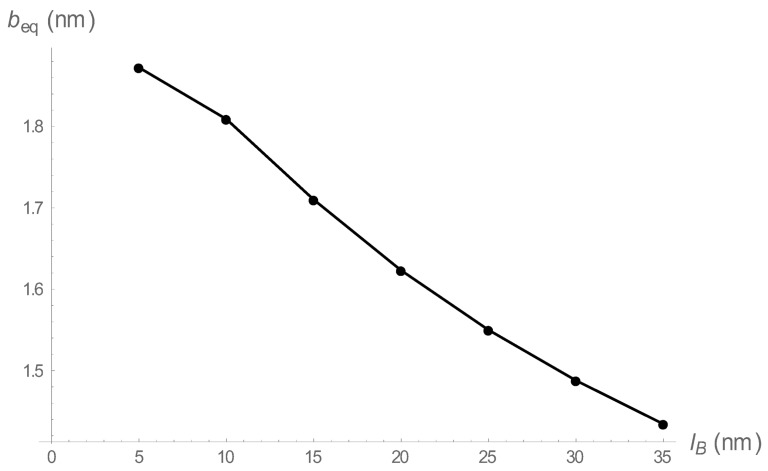
Equilibrium charge spacing as a function of Bjerrum length with spring constant *a* = 0.1 (nm)${}^{-2}$.

Figure 5 shows a plot of $b_{\mathrm{eq}}$ as a function of Coulomb strength (Bjerrum length) in the strong counterion condensation regime, extended far out to very high Bjerrum lengths (for comparison, $l_{B}$ is 0.71 nm for water and 1.7 nm for methanol, both at room temperature). Although condensation of counterions results in a contraction of the equilibrium bead-bead spacing, the effect in these poor solvents is slight, only about 25% in the range illustrated. The contribution of condensed counterions toward the free energy $g_{\mathrm{cc}}$ is relatively passive. These counterions are considered only to neutralize a fraction of the bare charge on the chain as they move more or less freely along its length. Evidently, if we wish to approach the collapse of polyelectrolyte gels observed in poorer solvents, we have to ascribe a more actionable role to the condensed counterions in such environments.

Polyelectrolyte gel collapse is usually attributed to attractive interactions among localized ion pairs or ionic multiplets [4]. My own preference is more generally to note that all ionic systems, ranging from dilute salt solutions up to crystals, necessarily exhibit the tendency for ions of one sign to be surrounded at various distance scales with ions of opposite sign. In the case of single-chain polyelectrolytes or polyelectrolyte segments in a gel, this tendency is manifested in the first place (if the bare charge density is high enough) by condensation of counterions, with little change in the conformation of the chains; but then in poorer solvents, the enhanced attraction promotes short-range quasi-crystal formation as the condensed counterions draw toward themselves the charged groups on the same or different polymer segments [18,20,21]. In fact, even in water, like-charged polyelectrolytes form loose clusters mediated (entropically) by condensed counterions [11].

In our present model of a one-dimensional bead-spring gel, we will assume that a condensed counterion can modify the spring constant if it is preferentially located between charged beads (think of a one-dimensional crystal), and thus, like the spring, provides a force resisting separation of the beads. In condensed counterion theory, the overall local concentration of condensed counterions, per bead, is $1/(8\pi el_{B}b^{2})$, where here “e” is the base of natural logarithms, not the unit charge [22]. In a cylindrical geometry, the subpopulation of condensed counterions that interact significantly with individual beads is contained in a volume $\pi l_{B}^{2}b$ per bead. Multiplying these two expressions gives us the number of condensed counterions per bead that can effectively act to strengthen the spring constant, $\left(1/8e\right)l_{B}/b$. We then assume that the spring constant is effectively strengthened by a proportional amount, and for the effective spring constant write the expression,

(3) $$a=a_{0}+\left(1/8e\right)t\left(l_{B}/b\right),$$

where $a_{0}$ is the spring constant in the absence of condensed counterions, and *t* is a free parameter (same units as $a_{0}$) expressing the contribution of a localized condensed counterion to the spring constant.

A more subtle consideration is that the locally active condensed counterions must be a subpopulation of all the condensed counterions. The radius *R* of the cylindrical volume that encloses the entire layer of condensed counterions is the square root of $8e\left[\left(l_{B}/b\right)-1\right]b^{2}$, and this radius must be larger than $l_{B}$. Our calculations must then be restricted to the range $1.05<l_{B}/b<20.7$, which presents no problem for physically realistic purposes.

Let us examine our expression Equation (3) more closely. It says among other things that the spring constant *a* depends on spring length *b*, so we have introduced nonlinearity into the elasticity. Further, it contains what others have called a cascading effect [4]. When *b* decreases, *a* increases, making the spring stiffer, more efficiently resisting stretching due to bead-bead Coulomb repulsion, thereby increasing the bead charge density, condensing more counterions, making the spring more stiff.

**Figure 6:**
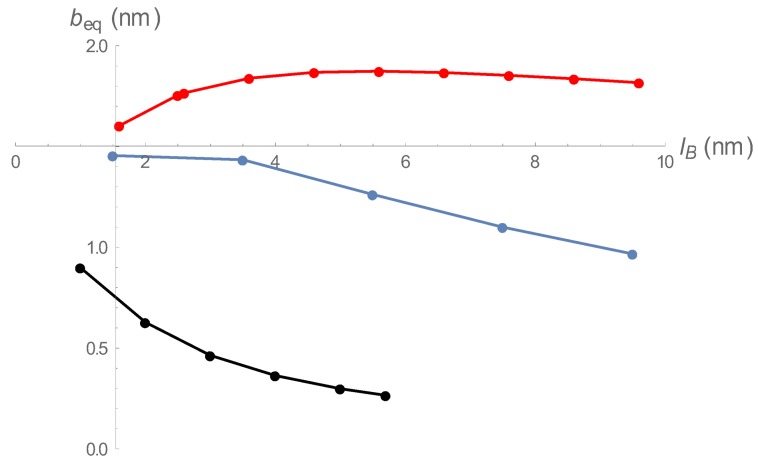
An illustration of the three ways that condensed counterions can affect contraction of the bead-spring chain: *t* = 0 nm${}^{-2}$ (red), *t* = 4 nm${}^{-2}$ (blue), *t* = 50 nm${}^{-2}$ (black).

In Figure 6 we show three scenarios. For counterion condensation with no effect on spring constant, t=0, and there is no significant shrinkage. For the relatively small value t=4 (nm)${}^{-2}$ the onset of counterion condensation at $l_{B}/b=1$ has little effect on contraction of charge spacing (spring length) until larger values of the Bjerrum length are achieved. However, for the larger value t=50 (nm)${}^{-2}$, the counterion stiffening effect dominates $a_{0}$ at the outset, and strong contraction begins right at the condensation threshold. These latter two types of “collapse” behavior have their counterparts in real physical systems (see Discussion section).

## 6. Discussion

To isolate two of the factors that combine to produce swelling and deswelling of polyelectrolyte gels, we have analyzed a linear bead-spring assembly, a “one-dimensional gel”, where the beads are identically charged, and the springs provide elastic resistance to stretching by Coulomb repulsion. The unstressed springs have zero length, and we use their equilibrium length $b_{\mathrm{eq}}>0$ as a measure of “swelling” of this one-dimensional model. In a previous paper [16], we restricted the bead density to values below the threshold for counterion condensation. Here, we extended the analysis to post-threshold densities. For both ranges we find significant deswelling (contraction) when the stiffness constant *a* for the spring increases, and significant swelling (increased stretch) when the ionic strength is lowered.

The properties of gels in solvents of varying quality has formed an important component of research in this field, allowing discovery of the iconic volume collapse transition of gels [2]. In our work we measure solvent quality by the Bjerrum length of the solvent, which is systematically increased by lowering the product of dielectric constant and temperature. Longer Bjerrum lengths signify enhanced Coulomb interactions in solvents poorer for salts and ionized macromolecules. On increasing the Bjerrum length we cross the threshold for counterion condensation on our one-dimensional gel. In a transition region we find gel-like behavior; the bead-spring assembly fluctuates between a contracted state with counterions condensed on the chain, and a stretched state from which counterions have been released. For a brief discussion of critical density fluctuations in gels, see Tanaka’s article in Reference [2].

On further increase of Bjerrum length the assembly contracts toward complete collapse. To obtain this gel-like behavior (some gels collapse progressively in some conditions, but conditions can be found for which nearly all gels collapse discontinuously), we introduced a variant on the “ion pairing in poor solvents” theme. An active fraction of condensed counterions, those close enough to the chain to attract adjacent beads, is assumed effectively to increase the spring constant. If this effect is absent the tendency toward collapse is insignificant. However, if the effect of condensed counterions on the spring constant dominates, the collapse becomes steeper and more cooperative.

To the best of our knowledge, there have been no attempts in the laboratory to correlate gel behavior with counterion condensation on the internal polymer skeleton. However, there have been both simulations and experiments on single-chain systems, that is, on free polyelectrolyte solutions. When a bead-spring assembly has been used in simulations of such systems, the spring constant is ordinarily set so high that the elastic response is effectively lost. Nonetheless, statistical ion pairing produces chain collapse, but only once the threshold for counterion condensation is exceeded [20,21]. The critical appearance of counterion condensation is manifest in the simulated data, and the shorter range effects inducing collapse are among counterions already condensed. These effects are quite absent in the range of solvent qualities where condensation of counterions is absent. Given that the interior of a polyelectrolyte gel resembles a polyelectrolyte solution, the more so the lighter the cross-linking, the results from single-chain simulations are likely not to be irrelevant for the interpretation of gel behavior [8].

The collapse of polyelectrolytes in poor solvents has also been studied experimentally. An especially interesting paper reports measurements of the behavior of poly(weak acids) in methanol [23]. It is clear enough that the collapse of poly(acrylic acid) and poly(methacrylic acid), both with Li${}^{+}$ and Na${}^{+}$ counterions, occur at or near the threshold for counterion condensation. The only apparent exception is LiPAA which collapses well on the far side of the condensation threshold. There is no reason to doubt the onset of Li${}^{+}$ condensation for this case as for the others. However, for reasons not currently understood, the lithium ions condensed on poly(acrylic acid) in methanol do not seem to form the specific short-range interactions leading to collapse until enough of them are present on the polymer chain. The *t* parameter describing them is not large, much as for the blue curve in Figure 6. Reasoning in this way encourages questions about the solvation of cations in methanol; about how the presence or absence of a methyl group on the polymer could influence its behavior in methanol or other solvents; or why the interaction of Li${}^{+}$ or Na${}^{+}$, once condensed on prototypical polyelectrolytes, such as PAA and PMA, might interact differently with carboxylate groups ionized in methanol compared with water. Questions like these would be of relevance also for gels made from the same polymers. One need not resort to poor solvents to collapse polyelectrolyte chains. NaPAA in water collapses on addition of Ca${}^{+2}$ ions, an observation of likely interest in gel studies [24,25].

It should be noted that the theory presented in this paper is on the mean-field level, which means that the solvent is considered as a continuum with dielectric properties, and the particles have no excluded volume. Molecular dynamics simulations on an atomistic level are not subject to these limitations, and recent results contain significant species-specific and solvation effects [26,27,28]. It also bears repetition that the present theory abstracts a gel as “one-dimensional,” thus does not consider interactions among separate ionized chains, which may be important in more realistic modeling. Water shows a significant decrease of the dielectric permittivity in confined geometries [29]. The associated increased Bjerrum length might encompass more than one chain even in a relatively dilute gel environment. Certainly there are avenues to be explored.

## References

[1] Smidsrød O., Moe S.T. (2008). Biopolymer Chemistry.

[2] Harland R.S., Prud’homme R.K. (1992). Polyelectrolyte Gels.

[3] Ottenbrite R.M.M., Park K., Okano T. (2010). BioMedical Applications of Hydrogels Handbook.

[4] Khokhlov A.R., Kramarenko E.Y. (1996). Weakly charged polyelectrolytes: Collapse induced by extra ionization. Macromolecules.

[5] Chen H., Calderer M.C., Mori Y. (2014). Analysis and simulation of a model of polyelectrolyte gel in one spatial dimension. Nonlinearity.

[6] Schneider S., Linse P. (2002). Swelling of cross-linked polyelectrolyte gels. Eur. Phys. J. E.

[7] Claudio G.C., Kremer K., Holm C. (2009). Comparison of a hydrogel model to the Poisson-Boltzmann cell model. J. Chem. Phys..

[8] Kobayashi H., Halver R., Sutmann G., Winkler R.G. (2017). Polymer conformations in ionic microgels in the presence of salt: Theoretical and mesoscale simulation results. Polymers.

[9] Denton A.R., Tang Q. (2016). Counterion-induced swelling of ionic microgels. J. Chem. Phys..

[10] Manning G.S. (2014). Excess counterion condensation on polyelectrolyte kinks and branch points and the interaction of skewed charged lines. Soft Matter.

[11] Manning G.S. (2011). Counterion condensation theory of attraction between like charges in the absence of multivalent counterions. Eur. Phys. J. E.

[12] Pietronave S., Arcesi L., D’Arrigo C., Perico A. (2008). Attraction between like-charge polyelectrolytes in the extended condensation theory. J. Phys. Chem. B.

[13] Tata B.V.R., Mohanty P.S., Valsakumar M.C. (2008). Bound pairs: Direct evidence for long-range attraction between like-charged colloids. Solid State Commun..

[14] Varghese A., Rajesh R., Vemparala S. (2012). Aggregation of rod-like polyelectrolye chains in the presence of monovalent counterions. J. Chem. Phys..

[15] Musheev M.U., Kanoatov M., Retif C., Krylov S.N. (2013). Stable DNA aggregation by removal of counterions. Anal. Chem..

[16] Manning G.S. (2018). A bead-spring chain as a one-dimensional polyelectrolyte gel. Soft Matter.

[17] Chahine J., Guimaraes M.A., Cavichiolli F.R. (1994). Temperature dependence of conformational properties of short polyelectrolytes from simulations at a single temperature. J. Phys. Chem..

[18] Stevens M.J., Plimpton S.J. (1998). The effect of added salt on polyelectrolyte structure. Eur. Phys. J. B.

[19] Manning G.S. (2002). Electrostatic free energy of the DNA double helix in counterion condensation theory. Biophys. Chem..

[20] Winkler M., Gold M., Reineker P. (1998). Collapse of Polyelectrolyte Macromolecules by Counterion Condensation and Ion Pair Formation: A Molecular Dynamics Simulation Study. Phys. Rev. Lett..

[21] Varghese A., Vemparala S., Rajesh R. (2011). Phase transitions of a single polyelectrolyte in a poor solvent with explicit counterions. J. Chem. Phys..

[22] Manning G.S. (1978). The molecular theory of polyelectrolyte solutions with applications to the electrostatic properties of polynucleotides. Q. Rev. Biophys..

[23] Pearsall S.K., Green M.M., Morawetz H. (2004). Titration of poly(carboxylic acid)s in methanol solution. Polymer chain extension, ionization equilibria, and conformational mobility. Macromolecules.

[24] Schweins R., Huber K. (2001). Collapse of sodium polyacrylate chains in calcium salt solutions. Eur. Phys. J. E.

[25] Schweins R., Lindner P., Huber K. (2003). Calcium induced shrinking of NaPA chains: A SANS investigation of single chain behavior. Macromolecules.

[26] Heyda J., Dzubiella J. (2012). Ion-specific counterion condensation on charged peptides. Soft Matter.

[27] Smiatek J., Wohlfarth A., Holm C. (2014). The solvation and ion condensation properties for sulfonated polyelectrolytes in different solvents. New J. Phys..

[28] Batys P., Luukkonen S., Sammalkorp M. (2017). Ability of the Poisson-Boltzmann equation to capture molecular dynamics predicted ion distribution around polyelectrolytes. Phys. Chem. Chem. Phys..

[29] Bonthuis D.J., Gekle S., Netz R.R. (2011). Dielectric profile of interfacial water and its effect on double-layer capacitance. Phys. Rev. Lett..

